# 

**Published:** 1837-10-01

**Authors:** 


### CONTENTS

OF THE

### MEDICO-CHIRURGICAL REVIEW,

### No. LIV. OCTOBER 1, 1837,

[BEING No. XIV. OF A DECENNIAL SERIES.]

### REVIEWS.

I.

I. Le Systeme Lymphatique. Par M. G. BRESCHET .. .. .. .. .. .. .. } 297

II. Nouvelles Recherches sur la Structure de la Peau. Par M. G. BRESCHET .. }

1. The Anatomy of the Lymphatic System .. .. .. .. .. .. .. .. .. .. 298

2. The Anatomy of the Skin .. .. .. .. .. .. .. .. .. .. .. .. .. 308

3. On the Diaphnogenous Apparatus, and on the Sudoriferous or Hidrophorous Canals .. .. .. .. .. .. .. .. .. .. .. .. 313

4. On the Cutaneous Apparatus of Inhalation .. .. .. .. .. .. .. .. .. 314

5. On the Blennogenous Apparatus—the Source of the Mucous Matter .. .. 316

A. The Blennogenous Apparatus .. .. .. .. .. .. .. .. .. .. 316

B. On the Chromatogenous Apparatus .. .. .. .. .. .. .. .. .. 317

C. Excreted Products of the Apparatus .. .. .. .. .. .. .. .. 317

6. On the Chromatogenous Apparatus, or Organs of the Secretion and Excretion of the colouring Matter .. .. .. .. .. .. .. .. .. .. .. .. 319

II.

Surgical Observations, with Cases and Operations. By J. C. WARREN, M.D. .. .. 322
1. Epidermoid Tumors .. .. .. .. .. .. .. .. .. .. .. .. .. .. 325
2. Dermoid Tumors.. .. .. .. .. .. .. .. .. .. .. .. .. .. .. 325
A. Lepoides . .. . .. .. .. .. .. .. .. .. .. .. .. .. .. 325
B. Keloides .. .. .. .. .. .. .. .. .. .. .. .. .. .. .. 326
C. Eiloides .. .. .. .. .. .. .. .. .. .. .. .. .. .. .. 327
3. Tumors of the Cellular Membrane .. .. .. .. .. .. .. .. .. .. 327
4. Muscular Tumors .. .. .. .. .. .. .. .. . .. .. .. . .. 328
A. Encysted Muscular Melanosis .. .. .. .. .. .. .. .. .. .. 329
B. Scirrhous Muscular Tumor .. .. .. .. .. .. .. .. .. .. .. 329
C. Tumors of the Tendons .. .. .. .. .. .. .. .. .. .. .. 332
5. Periosteal Tumors .. .. .. .. .. .. .. .. .. .. .. .. .. 335
6. Osseous Tumors .. .. .. .. .. .. .. .. .. .. .. .. .. .. 336
A. Osteo-Sarcoma .. .. .. .. .. .. .. .. .. .. .. .. .. .. 338

III.

I. Medico-Chirurgical Transactions. Vol. XX. .. .. .. .. .. .. .. } 345
II. The Transactions of the Provincial Medical and Surgical Association. Vol. V. }
III. Elements of Medicine. Vol. I. On Morbid Poisons. By R. WILLIAMS, M.D. }
1. Observations on Vascular Appearances of Mucous and Serous Membranes, as indicative of Inflammation. By J. YELLOLY, M.D... .. .. .. .. 345
2. On the Effects of Morbid Poisons .. .. .. .. .. .. .. .. .. .. 351
A. On the Treatment of Injuries received in Dissection. By R. A. STAFFORD, Esq. .. .. .. .. .. .. .. .. .. .. .. .. .. .. 351

B. Case of Glanders in the Human Subject. By JAMES JOHNSTONE, M.D. 359
C. On the Poison of Measles. By R. WILLIAMS, M.D. .. .. .. .. .. 361
D. On the Poison of Erysipelas .. .. .. .. .. .. .. .. .. .. .. .. .. 364
3. On the Use of the Bromide of Potassium in Diseases of the Spleen .. .. 370
4. Case in which the Memory of Language was lost. By T. SHAPTER, M.D. .. 372

IV.

Observations on the Topography, Climate, and Prevalent Diseases of the Island of Jersey. By G. S. HOOPER, M.D. .. .. .. .. .. .. .. .. .. .. .. .. 377
1. Diseases of the Island .. .. .. .. .. .. .. .. .. .. .. .. .. .. .. 377

V.

ON THE USE OF MERCURY IN THE VENEREAL DISEASE.

I. Practical Observations on the Venereal Disease, and on the Use of Mercury. By ABRAHAM COLLES, M.D. .. .. .. .. .. .. .. .. .. .. .. ..
II. The Works of John Hunter, F.R.S. Edited by J. F. PALMER. Vol. II. ..
III. Historical and Medical Researches into the Origin, Nature, and Treatment of Syphilis. By M. DEVERGIE .. .. .. .. .. .. .. . .. .. .. .. } 379
1. Natural History of Syphilis .. .. .. .. .. .. .. .. .. .. .. .. . 390
2. On Chancre .. .. .. .. .. .. .. .. .. .. .. .. .. .. .. .. .. 397

VI.

RECENT WORKS ON THE PRACTICE OF MEDICINE.

I. Principles of the Theory and Practice of Medicine. By MARSHALL HALL, M.D.
II. Elements of the Practice of Physic. By D. CRAIGIE, M.D. .. .. .. .. } 403

VII.

The Spas of Germany. By Dr. GRANVILLE .. .. .. .. .. .. .. .. .. .. .. 422
1. Baden Baden, 423—2. Wildbad, 424—3. Bad Gastein, 426—4. Carlsbad, 428—5. Marienbad, 432—6. Egra, 434—7. Pullna, 436—8. Tœplitz, 436—9. Leibenstein, 437—10. Kissengen, 437—11. Weisbaden, 438—12. Schlangenbad, 439—13. Schwalbach, 440—14. Ems, 440.

VIII.

VEGETABLE MORPHOLOGY.

I. An Introduction to Botany. By JOHN LINDLEY, F.R.S. .. .. .. .. ..
II. Nouveau Systeme de Physiologie Vegetale et de Botanique Par F. V. RASPAIL } 442

IX.

TYPHOID FEVER.

I. Clinique Medicale de l'Hôpital de la Charité. Par M. BOUILLAUD.. .. ..

II. Anatomical, Pathological, and Therapeutic Researches on Gastro-Enterite. By P. C. LOUIS .. .. .. .. .. .. .. .. .. .. .. .. .. .. } 450

### PERISCOPE.

### Bibliographical Notices.

1. The Cyclopædiæ.

1. The Cyclopædia of Practical Surgery. Edited by W. B. Costello, M.D. Part II. 459

2. The Cyclopædia of Anatomy and Physiology. Edited by R. Todd, M.D. Part XI. 464

A. New Mode of Treatment for Ulcers and Granulating Wounds. By F. C. Skey, F.R.S. .. .. .. .. .. .. .. .. .. .. .. .. .. .. 466

2. Natural History.
1. A History of British Quadrupeds. By Thomas Bell, F.R.S. .. .. .. .. 468
2. A History of British Birds. By W. Yarrell, F.L.S. .. .. .. .. .. .. .. 468
3. On the Nature and Treatment of Diseases of the Heart. By J. Wardrop, M.D. .. 469

### Spirit of the British and American Periodicals.

1. The present State of Pharmacy in Germany .. .. .. .. .. .. .. .. .. .. .. 473
2. Case of Hernia Pericardii. By T. Hart, M.D. .. .. .. .. .. .. .. .. .. 476
3. On the Possibility of transplanting the Cornea for Blindness. By Dr. Bigger .. 477
4. On violent Pulsations in the Epigastric Region. By W. Faussett, A.B. .. .. 480
5. Medicine in America .. .. .. .. .. .. .. .. .. .. .. .. .. .. .. .. 482
1. Cases of Aneurysm cured by Operation. By Dr. Morisson .. .. .. .. .. 482
A. Femoral Aneurysm .. .. .. .. .. .. .. .. .. .. .. .. .. .. 482
B. Successful Operation beyond the Aneurysm .. .. .. .. .. .. .. .. 482
2. Observations on the Treatment of Puerperal Convulsions. By W. Denny, M.D. 485
3. Supposed new view of certain Dislocations. By S. Annan, M.D. .. .. .. .. 487
A. Reduction of a Dislocation of the Head of the Humerus into the Axilla .. 487
B. New Mode of reducing a Dislocation into the Ischiatic Notches .. .. 488
4. Successful Ligature of the Internal Iliac Artery. By V. Mott, M.D. .. .. 490
5. Case of Congenital Enlargement of the Tongue removed by Operation. By T. Harris, M.D. .. .. .. .. .. .. .. .. .. .. .. .. .. .. .. 490
6. Delivery of a Fœtus through the Abdominal Parietes. By S. H. Harris, M.D. 492
7. Diseases of the Sandwich Islands .. .. .. .. .. .. .. .. .. .. .. .. 493
8. Case of Worms in the Urinary Bladder .. .. .. .. .. .. .. .. .. .. 495
6. Preparation of Phloridzine .. .. .. .. .. .. .. .. .. .. .. .. .. .. 495
7. Opinions of Napoleon on Medical Education .. .. .. .. .. .. .. .. .. 496
8. Report on the National Vaccine Establishment.. .. .. .. .. .. .. .. 497
9. Vicarious Menstruation from the Skin of the Thorax .. .. .. .. .. .. .. 497
10. Substitute for the Liquor Opii Sedativus of Battley .. .. .. .. .. .. .. 498
11. On poisoning with the Esculent Mushroom. By D. Edwards, Esq. .. .. . 498
12. Cure of Club Foot by Division of the Tendo Achillis .. .. .. .. .. .. 499
13. Spontaneous Cure of a gelatinous Polypus Nasi .. .. .. .. .. .. .. .. 500
14. Opinions on the Contagion of Glanders .. .. .. .. .. .. .. .. .. .. .. 500
15. Dr. Graves on the Pathology of Phlegmasia Dolens .. .. .. .. .. .. .. .. 500

### Spirit of the Foreign Periodicals, &c.

1. Memoir on some remarkable Cases of Organic Anomalies .. .. .. .. .. .. 502
A. Urinary Apparatus .. .. .. .. .. .. .. .. .. .. .. .. .. .. .. 503
B. Generative Apparatus. .. .. .. .. .. .. .. .. .. .. .. .. .. .. .. 504
C. Lateral Transposition of the Thoracic and Abdominal Viscera .. .. .. 506
2. Psychological Physiology.
1. Relation of the Weight of the Cerebral Mass with the Development of Intelligence .. .. .. .. .. .. .. .. .. .. .. .. .. .. .. .. .. 507
3. On the Treatment of Typhoid Fever by Purgatives .. .. .. .. .. .. .. .. 510
4. Discussion on the Nature and Treatment of Glanders in Man.. .. .. .. .. .. 518
5. On the Endermic and Inoculative Method of Treatment .. .. .. .. .. .. .. 524
6. On the Action of certain Medicines on the Heart .. .. .. .. .. .. .. .. 526
A. Assafœtida .. .. .. .. .. .. .. .. .. .. .. .. .. .. .. .. .. 526
B. Camphor .. .. .. .. .. .. .. .. .. .. .. .. .. .. .. .. .. .. 526
C. Digitalis .. .. .. .. .. .. .. .. .. .. .. .. .. .. .. .. .. 527
D. Polygala Senega .. .. .. .. .. .. .. .. .. .. .. .. .. .. .. 527
7. Observations on the Cancrum Oris of Children .. .. .. .. .. .. .. .. 527
8. General Remarks on the Diseases of Children. By M. Baudelocque .. .. .. 530
9. On the Treatment of Gastralgia. By Dr. Lombard .. .. .. .. .. .. .. 532
10. Case of Abdominal Tumor which weighed 120 pounds .. .. .. .. .. .. .. 533
11. Ascites cured with the Inunction of Veratria Ointment .. .. .. .. .. .. 535
12. Report of M. Desportes on the Salpetriere and Bicetre Lunatic Hospitals .. .. 535
13. Statistical Reports of Hospitals .. .. .. .. .. .. .. .. .. .. .. .. 539
14. Case of confirmed Insanity cured by a sudden Fright .. .. .. .. .. .. 540

15. Cases of hæmorrhagic Diathesis. By Professor Kuhl .. .. .. .. .. .. .. 540
16. Two Cases, in which it is alleged that a Lizard was vomited from the Intestines .. 541
17. Employment of a Stream of Cold Water, in Cases of Wounds and other Injuries .. 543
18. Reduction of an old Dislocation of a Shoulder—Death the same day .. .. .. 545
19. Curious Case of simultaneous Dislocation of both Thighs .. .. .. .. .. .. 546
20. Variety of Club-foot treated by Division of the Tendo Achillis .. .. .. .. 546
21. Wry-neck—Section of the Sterno-mastoid Muscle .. .. .. .. .. .. .. .. .. 548
22. Successful Case of Amputation at the Hip-joint .. .. .. .. .. .. .. .. 549
23. Cases of Amputation .. .. .. .. .. .. .. .. .. .. .. .. .. .. .. 550
24. Radical Cure of Varicose Veins and of Herniæ by Acupuncturation .. .. .. 551
25. Spontaneous Gangrene of the Leg—Amputation before the Line of Separation was established.. .. .. .. .. .. .. .. .. .. .. .. .. .. 552

### Clinical Review, and Hospital Reports.

1. Fever in America and Ireland.

PHILADELPHIA HOSPITAL.

1. Difference between Dothinenteritis and the Typhus Fever which occurred at Philadelphia in 1836. By Dr. Gerhard .. .. .. .. .. .. .. .. 553

ST. JOHN'S FEVER HOSPITAL.

2. Report of St. John's Fever and Lock Hospitals, Limerick. By Dr. Geary .. 557

NAVAN FEVER HOSPITAL.

3. Observations on Certain Remedies in Typhus Fever. By Dr. Hudson.. .. 559
4. On the Cause of Tympanitic Sound on Percussion over an inflamed Lung. By Dr. Hudson .. .. .. .. .. .. .. .. .. .. .. .. .. .. 561

ST. GEORGE'S HOSPITAL.

2. Quarterly Report of Patients treated in St. George's Hospital. By Dr. Seymour 562

### Miscellanies.

1. British Association.. .. .. .. .. .. .. .. .. .. .. .. .. .. .. .. 569

1. Section E.—Anatomy and Medicine .. .. .. .. .. .. .. .. .. .. .. .. 570

A. On the Motions and Sounds of the Heart, 2nd Report.. .. .. .. .. .. 571

B. On the Physical and Chemical Characters of Expectoration in different Diseases of the Lungs. By Mr. Brett .. .. .. .. .. .. .. .. .. 573

C. On the Cause of Death from a Blow on the Stomach. By Dr. Holland .. 576

D. Experimental Investigation into the Glosso-pharyngeal, Pneumogastric, and Spinal Accessory Nerves. By Dr. John Reid .. .. .. .. .. .. 578

E. Observations on the Structure of the Sacrum in Man and some of the Lower Classes of Animals. By Mr. Carlisle.. .. .. .. .. .. .. .. 580

F. On the Epidemic Cholera By Dr. Black .. .. .. .. .. .. .. .. .. 580

G. Dysmenorrhea. By Dr. Macintosh .. .. .. .. .. .. .. .. .. .. 582

H. Experiments on the Connexion between Nerves and Muscles. By Dr. Madden 583

I. On the Order of the Succession of the Motions of the Heart. By Dr. O'Bryan Bellingham.. .. .. .. .. .. .. .. .. .. .. .. .. .. 583

J. Observations on the Disease called Cocobâe by the Africans. By Dr. J. Hancock .. .. .. .. .. .. .. .. .. .. .. .. .. .. .. .. 584

2. Transposition of the Viscera.. .. .. .. .. .. .. .. .. .. .. .. .. 584

3. Sudden and Fatal Hæmoptysis .. .. .. .. .. .. .. .. .. .. .. .. 585

### Obituary.

Death of Mr. Lynn .. .. .. .. .. .. .. .. .. .. .. .. .. .. .. .. 586
Death of Rasori .. .. .. .. .. .. .. .. .. .. .. .. .. .. .. .. 586

BIBLIOGRAPHICAL RECORD .. .. .. .. .. .. .. .. .. .. .. .. .. .. 587

### Extra-Limites.

PHYSIOLOGICAL EFFECTS OF RAIL-ROAD TRAVELLING.

The Railroad Steamer, By Dr. James Johnson .. .. .. .. .. .. .. .. .. 589

